# Consolidation whole abdomen irradiation following adjuvant carboplatin-paclitaxel based chemotherapy for advanced uterine epithelial cancer: feasibility, toxicity and outcomes

**DOI:** 10.1186/1748-717X-8-236

**Published:** 2013-10-14

**Authors:** Nathalie Rochet, Rachel S Kahn, Andrzej Niemierko, Thomas F Delaney, Anthony H Russell

**Affiliations:** 1 Department of Radiation Oncology, Massachusetts General Hospital - Harvard Medical School, 100 Blossom Street, COX3, Boston, MA 02114, USA; 2 Department of Radiation Oncology, University of Heidelberg, INF 400, Heidelberg 69120, Germany; 3 Brown University, Warren Alpert Medical School, Providence, RI, USA; 4 Permanent address: Department of Radiation Oncology, Lausanne University Hospital, Centre Hospitalier Universitaire Vaudois, Rue du Bugnon 21, Lausanne, Vaud CH-1011, Switzerland

**Keywords:** Whole abdomen irradiation, Advanced endometrial cancer, Chemotherapy, Radiotherapy

## Abstract

**Background:**

To evaluate feasibility and preliminary outcomes associated with sequential whole abdomen irradiation (WAI) as consolidative treatment following comprehensive surgery and systemic chemotherapy for advanced endometrial cancer.

**Methods:**

We conducted a retrospective analysis of patients treated at our institution from 2000 to 2011. Inclusion criteria were stage III-IV endometrial cancer patients with histological proof of one or more sites of extra-uterine abdomen-confined disease, treated with WAI as part of multimodal therapy. Endpoints were feasibility, acute toxicity, late effects, recurrence-free survival (RFS) and overall survival (OS). Twenty patients were identified. Chemotherapy consisted of 3 to 6 cycles of a platinum-paclitaxel regimen in 18 patients. WAI was delivered using conventional technique to a median total dose of 27.5 Gy.

**Results:**

No grade 4 toxicities occurred during chemotherapy or radiotherapy. No radiation dose reduction was necessary. Three patients developed small bowel obstruction, all in the context of recurrent intraperitoneal disease. Kaplan-Meier estimates and 95% confidence intervals for RFS and OS at one year were 63% (38–80%) and 83% (56-94%) and at 3 years 57% (33-76%) and 62% (34-81%), respectively. On univariate Cox analysis, stage IVB and serous papillary (SP) histology were found to be statistically significantly (at the p = 0.05 level) associated with worse RFS and OS. The peritoneal cavity was the most frequent site of initial failure.

**Conclusions:**

Consolidative WAI following chemotherapy is feasible and can be performed without interruption with manageable acute and late toxicity. Patients with endometrioid adenocarcinoma, especially stage FIGO III, had favorable outcomes possibly meriting prospective evaluation of the addition of WAI following chemotherapy in selected patients. Patients with SP do poorly and do not routinely benefit from this approach.

## Background

Endometrial cancer (EC) is the most common malignancy of the female genital tract in the United States with an estimated 47,100 new cases and 8,000 deaths in 2012 [1]. Most patients are diagnosed with early stage disease and have an excellent prognosis. However, for the <20% of patients presenting with extra-uterine disease (stages III and IV), the 5-year overall survival (OS) rates decrease dramatically and range from 30-89% to 0-20%, respectively [2-5]. Optimal management for these patients remains undefined.

Gynecologic Oncology Group (GOG) protocol 122 prospectively randomized patients between chemotherapy and whole abdomen irradiation (WAI), and established the superiority of systemic chemotherapy [5]. As a consequence, WAI is generally no longer recommended in the postoperative treatment of patients with stage III or IV disease. However, recurrences were frequent in both treatment arms of GOG 122. More than half of the patients progressed or failed in the abdomen illustrating the need for further therapeutic improvements in this high-risk patient population.

A predilection for diffuse peritoneal spread is the rationale for the use of whole abdomen irradiation (WAI), which appeared to have provided therapeutic benefit in both retrospective and prospective studies including patients with proven peritoneal dissemination [5-10]. It is logical to speculate that adding WAI following chemotherapy may result in superior survival outcomes by improving regional control. Recent studies have reported encouraging preliminary results of multimodal treatment, but have included few patients treated with WAI [11-14].

We sought to review outcomes of WAI as part of multimodal post surgical treatment of women with abdomen-confined advanced EC at Massachusetts General Hospital (MGH).

## Methods

### Patient identification and data collection

After obtaining approval from the MGH Institutional Review Board, we conducted a single-institution retrospective review of feasibility, toxicity and outcomes. Patients were identified using our tumor registry to meet following the inclusion criteria: patients with locally advanced histologically confirmed EC with histologic proof of one or more sites of abdomen-confined extra-uterine disease, who underwent primary surgery followed by multimodal chemotherapy and radiotherapy including WAI between 2000 and 2011.

Exclusion criteria were the presence of known residual macroscopic disease at the time of consolidation WAI, the presence of metastasis beyond the abdomen, recurrence after prior treatment or a different histology than endometrioid adenocarcimona (EAC), serous papillary (SP) or clear cell (CC) carcinoma. Patients were also excluded if they had a FIGO 1988 stage IIIA disease based on positive cytology only.

Complete clinical data were abstracted by review of operative notes, and both hospital and outpatient chart. Toxicity was assessed using Common Terminology Criteria for Adverse Events CTCAE version 4.03 (2010).

### Consent

Written informed consent was obtained from the patients for the publication of this report.

### Patient and treatment characteristics

A total of 20 patients were included in the study. Patient and treatment characteristics are listed in Table 1. Primary surgery procedure included comprehensive surgical staging and cytoreductive surgery with at least total abdominal hysterectomy, bilateral salpingo-oophorectomy, debulking of gross disease and peritoneal cytology in all patients. Lymph node dissection or sampling was performed in 17 patients (85%), either pelvic only (n = 8) or both pelvic and para-aortic (n = 9). Workup for disease extent included a CT-scan of the pelvis, abdomen and chest for all patients.

**Table 1:** Patients’ and treatment characteristics (n = 20)

**Category**	**Distribution and%**
Age (median, range)	61 (34–77)
2009 FIGO stage	
IIIA	7 (35%)
IIIC	5 (25%)
IIIC1	2 (10%)
IIIC2	3 (15%)
IVB	7 (35%)
No classification available *	1 (5%)
Histology	
Endometrioid (pure)	13 (65%)
Papillary serous (pure)	4 (20%)
Papillary serous (mixed)	3 (15%)
Grade	
1	3 (15%)
2	7 (35%)
3	10 (50%)
Nodal status	
pN0	11 (55%)
pN+	6 (30%)
pNx	3 (15%)
Peritoneal cytology	
Positive or suspicious for malignant cells	15 (75%)
Negative	4 (20%)
Not done	1 (5%)
**SURGERY**	
Surgical approach	
Laparotomy	15 (75%)
Laparoscopy	5 (25%)
Lymph node dissection performed	17 (85%)
Pelvic only	8 (40%)
Pelvic + PA	9 (45%)
Total number of dissected LN (median, range)	18 (3–46)
**CHEMOTHERAPY**	
Number of cycles (received/planned)	
0/6 cycles	2 (10%)
3/3 cycles	2 (10%)
4/6 cycles	1 (5%)
5/6 cycles	1 (5%)
6/6 cycles	14 (70%)
Drug combination (n = 18)	
TP	14 (78%)
TAP	4 (22%)
**RADIOTHERAPY**	
WAI total dose (median, range)	
Total dose (Gy)	27.5 (20–30)
Daily dose (Gy)	1.25 (1.0 - 1.25
Additional volume (boost)	
Pelvic only	7 (35%)
Pelvic + PA	2 (10%)
Cumulative total dose(median, range)	
Pelvis (Gy)	46.7 (44.8 - 48)
PA (Gy)	46.7 (44.8 - 55.7)
Vaginal cuff brachytherapy	17 (85%)
Dose (median, range)	
Total dose (Gy)	18 (13–21)
Number of fractions	3 (3–5)
**TIMING** (median, range)	
Between surgery and chemo start (days)	24 (9–48)
Between end of chemo and RT start (days)	48 (21–104)
Between surgery and completion of all treatments (months)	5.7 (1.5 - 7.6)
Duration of RT (elapsed days)	35 (11–55)

*multifocal cancer arising from endometriosis.

*Abbreviations*: *Chemo* Chemotherapy, *LN* Lymph nodes, *PA* Para-aortic, *RT* Radiotherapy, *TP* Paclitaxel (175 mg/m^2^) and Carboplatin (Area under the Curve [AUC] 5); TAP = Paclitaxel (175 mg/m^2^), Doxorubicin (45 mg/m_2_) and Carboplatin (AUC5).

Adjuvant chemotherapy as part of a multimodal treatment was recommended in all 20 patients by a multidisciplinary tumor board. However, 2 patients did not receive chemotherapy for the following reasons: patient refusal (n = 1) and medical contraindications (n = 1) in a patient with severe diabetes and organ damage. Both were included in this study as an intent-to-treat analysis. Adjuvant chemotherapy consisted of 3 to 6 cycles intravenous Paclitaxel (T) and Carboplatin (P) based chemotherapy on a q21 day schedule.

A total of 5 patients had postoperative macroscopic residual disease. None of the patients had demonstrable macroscopic residual disease at the time of radiotherapy. Three patients had been considered sub optimally debulked in the surgical report. After completion of chemotherapy, they underwent a 2^nd^-look laparoscopy that showed no evidence of persistent disease in one case and microscopic foci of residual disease in two cases. Two patients considered as optimally debulked had [18 F]-fluorodeoxyglucose (FDG) uptakes compatible with residual disease in a postoperative FDG-Positron Emission Tomography/Computed Tomography (PET/CT) using contrast enhanced diagnostic quality CT. After completion of chemotherapy, subsequent PET/CT scan showed near complete resolution of the areas of abnormal uptake.

WAI was administered following completion of chemotherapy and after hematologic toxicity recovered to an acceptable level. Median time between between completion of chemotherapy and onset of radiotherapy was 48 days (range, 21–104 days). The whole abdomen was treated based on a computer tomography simulation using anterior-posterior/postero-anterior (ap/pa) extended skin surface distance (SSD) opposed fields (6 to 18 MV photons) to a total dose ranging from 20 to 30 Gy (median 27.5). The daily fraction size was 1.0 -1.25 Gy, 5 days per week using 6 to 18 MV photons. One patient was treated 1.0 Gy twice-a-day (b.i.d.). Kidneys and liver shielding was used to maintain the total dose ≤18 Gy for the kidneys and ≤22 Gy for the liver.

Eleven patients (55%) received a subsequent pelvic boost volume (boost), which was combined with an extended para-aortic field in 9 cases (45%). Indication for a boost was: the presence of nodal metastases (n = 6), stage IVB disease with aggressive histology and unknown nodal status (n = 1), postoperative PET-positive residual pelvic lymph node (n = 1) and stage IVB disease in a patient who had refused chemotherapy (n = 1). Pelvic and para-aortic boosts were given with 2D or 3D planned ap/pa opposed fields using 6–23 MV photons to a median total dose of 19.2 Gy (range, 18.0 – 28.3) in 1.6 to 1.8 Gy daily fractions. Two patients with para-aortic nodal involvement were given additional involved-field boost to the para-aortic area at risk. The total cumulative external beam doses to the pelvis ranged between 44.8 to 48 Gy (median 46.7 Gy) and between 44.8 to 55.7 Gy for the para-aortic region (median 46.7 Gy). One patient received the involved field boost treatment prior to WAI because of concerns regarding hematologic tolerance. All patients except one received the entire radiotherapy treatment at MGH affiliated facilities.

An additional Ir192-High Dose Rate vaginal cuff brachytherapy was performed in 17 cases to a median total dose of 18 Gy (range, 13–21 Gy) in a median of 3 fractions (range, 3–5 fractions). Dose was prescribed to the applicator surface.

Upon treatment completion patients were evaluated at regular intervals at MGH facilities. Imaging was performed in any patient with current recurrence suspected on the basis of symptoms or physical examination, but was not routinely performed in asymptomatic patients with normal physical examination.

### Statistics

Recurrence-free survival (RFS) and OS estimates were calculated by Kaplan-Meier (KM) actuarial method from date of completion of all treatments and reported for 1, 3 and 5 years. OS times were calculated to date of death or were censored at date of last documented contact for patients still alive at time of analysis. RFS times were calculated to date of first known progression or death, or were censored at the date of last contact for patients still alive and who have not progressed.

Univariate Cox analysis was performed for following covariates: age at diagnosis (continuous variable), histology (EAC vs. SP), stage of disease (III vs. IVB), pelvic and/or para-aortic nodal involvement (yes vs. no), presence of residual disease after surgery, presence of residual disease after chemotherapy, completion of chemotherapy (complete and full dose vs. incomplete or reduced dose), pelvic boost (yes vs. no), para-aortic boost (yes vs. no), WAI total dose (continuous variable), elapsed time between end of chemotherapy and start of radiotherapy (continuous variable). Significance was defined as a *p* value <0.05.

All analyses were performed using StataCorp. 2011 (*Stata Statistical Software: Release 12*. College Station, TX: StataCorp LP).

## Results

### Feasibility and acute toxicity

No CTCAE grade 4 side effects were observed during chemotherapy or radiotherapy. Overall hematologic toxicity and elevation of liver enzymes are summarized in Table 2. During chemotherapy, 4 patients required some dose reduction for the following reasons: CTC grade 3 neuropathy and/or myalgia (n = 2) and CTC grade 3 febrile neutropenia (n = 2). Two patients required blood transfusions and 6 required Granulocyte-Colony Stimulating Factor (G-CSF) during chemotherapy but none required G-CSF during radiotherapy. Overall, acute toxicity was manageable with most patients experiencing CTCAE grade 1–2 fatigue, neuropathy and/or nausea during chemotherapy and CTCAE grade 1–2 nausea and/or diarrhea that responded to symptomatic medication during radiotherapy.

**Table 2:** Hematologic toxicities (nadir) and elevation of liver enzymes (peak) during treatment

	**N/A**	**Grade 0**	**Grade 1**	**Grade 2**	**Grade 3**	**Grade 4**
During chemotherapy (n = 18)						
Elevation ALT	4 (22%)	9 (50%)	5 (28%)	0	0	0
Elevation AST	5 (28%)	10 (56%)	3 (17%)	0	0	0
Anemia (Hgb)	2 (11%)	3 (17%)	9 (50%)	4 (22%)	0	0
Thrombocytopenia	2 (11%)	13 (72%)	2 (11%)	1 (6%)	0	0
Neutropenia	2 (11%)	12 (67%)	0	2 (11%)	2 (11%)*	0
During RT (n = 20)						
Elevation ALT	5 (25%)	11	4 (20%)	2 (10%)	0	0
Elevation AST	5 (25%)	11	4 (20%)	0	0	0
Anemia (Hgb)	0	3 (15%)	10 (50%)	6 (30%)	1 (5%)	0
Thrombocytopenia	0	5 (25%)	10 (50%)	4 (20%)	1(5%)	0
Neutropenia	2 (10%)	6 (30%)	6 (30%)	4 (20%)	2 (10%)	0
Hypokalemia	6 (30%)	6 (30%)	1 (5%)	3 (15%)	4 (20%)	0
6 weeks after RT (n = 20)						
Elevation ALT	9 (45%)	5 (25%)	5 (25%)	0	1 (5%)	0
Elevation AST	9 (45%)	5 (25%)	5 (25%)	0	1 (5%)	0
Anemia (Hgb)	6 (30%)	3 (15%)	8 (40%)	3 (15%)	0	0
Thrombocytopenia	6 (30%)	7 (35%)	5 (25%)	1 (5%)	1 (5%)	0
Neutropenia	7 (35%)	9 (45%)	1 (5%)	2 (10%)	1 (5%)	0

Common terminology criteria for adverse events (CTCAE) version 4.03 (2010).

* febrile neutropenia.

*Abbreviations*: *N/A* Information not available at time of analysis, *RT* Radiotherapy; *Hgb* Hemoglobin, *ALT* Alanine aminotransferase, *AST* Aspartate aminotransferase.

Radiotherapy could be completed as planned in all the patients without toxicity-related interruptions or unacceptable acute toxicity and was performed on an exclusively outpatient basis in 85% of the cases. Three patients needed a brief hospitalization for supportive care and observation for the following reasons: twice daily 1.0 Gy WAI (n = 1), acute radiation enteritis (n = 1), CTCAE grade 3 anemia requiring blood transfusion and dehydration (n = 1). Electrolyte replenishment was given in seven patients for hypokalemia (oral or IV).

### Late toxicity

All late adverse events are presented in Table 3. No cancer-free patient has developed late bowel toxicity > CTC 2. Three patients developed subsequent small bowel obstruction with 2 requiring surgery and another patient experienced a sigmoid perforation with florid peritonitis, all in the context of recurrent intraperitoneal disease. Fifty percent of the patients experienced a transient asymptomatic elevation of transaminases with only one CTCAE grade 3.

**Table 3:** Late adverse events (n = 20)

**Events**	**Distribution and%**
Small bowel obstruction *	3 (15%)
Sigmoid perforation*	1 (5%)
Chronic radiation enteritis and proctitis CTC 1	1 (5%)
Radiation pneumonitis CTC 1	1 (5%)
Vaginal dryness and teleangiectasia	3 (15%)
Dyspareunia	1 (5%)
Urinary urgency CTC 1	1 (5%)
Chronic fatigue	3 (15%)
Chronic neuropathy CTC 2	2 (10%)
Chronic leg oedema	1 (5%)
Tight cellulitis	1 (5%)
Infected lymphocele	1 (5%)
Renal failure**	1 (5%)
Erosive gastritis	1 (5%)

* all in the context of recurrent intraperitoneal recurrent disease.

**Attributed to severe type II diabetic nephropathy, did not receive chemo because of severe diabetes.

Some patients have >1 adverse event.

### Outcomes

The median observation time from completion of all treatments to death or to the end of the observation period (January 2012) was 21.5 months (range, 1.7 months-11.5 years). At the time of analysis, 13 patients were alive and 7 had died. Twelve patients were alive with no evidence of disease at time of last follow-up and one patient was alive with progression of disease. Median follow up for surviving patients was 31.2 months (range, 3.9 months – 11.5 years). Six patients died of disease. One patient died of intercurrent illness (pontine hemorrhage) and the autopsy showed no evidence of cancer.

**Figure 1:**
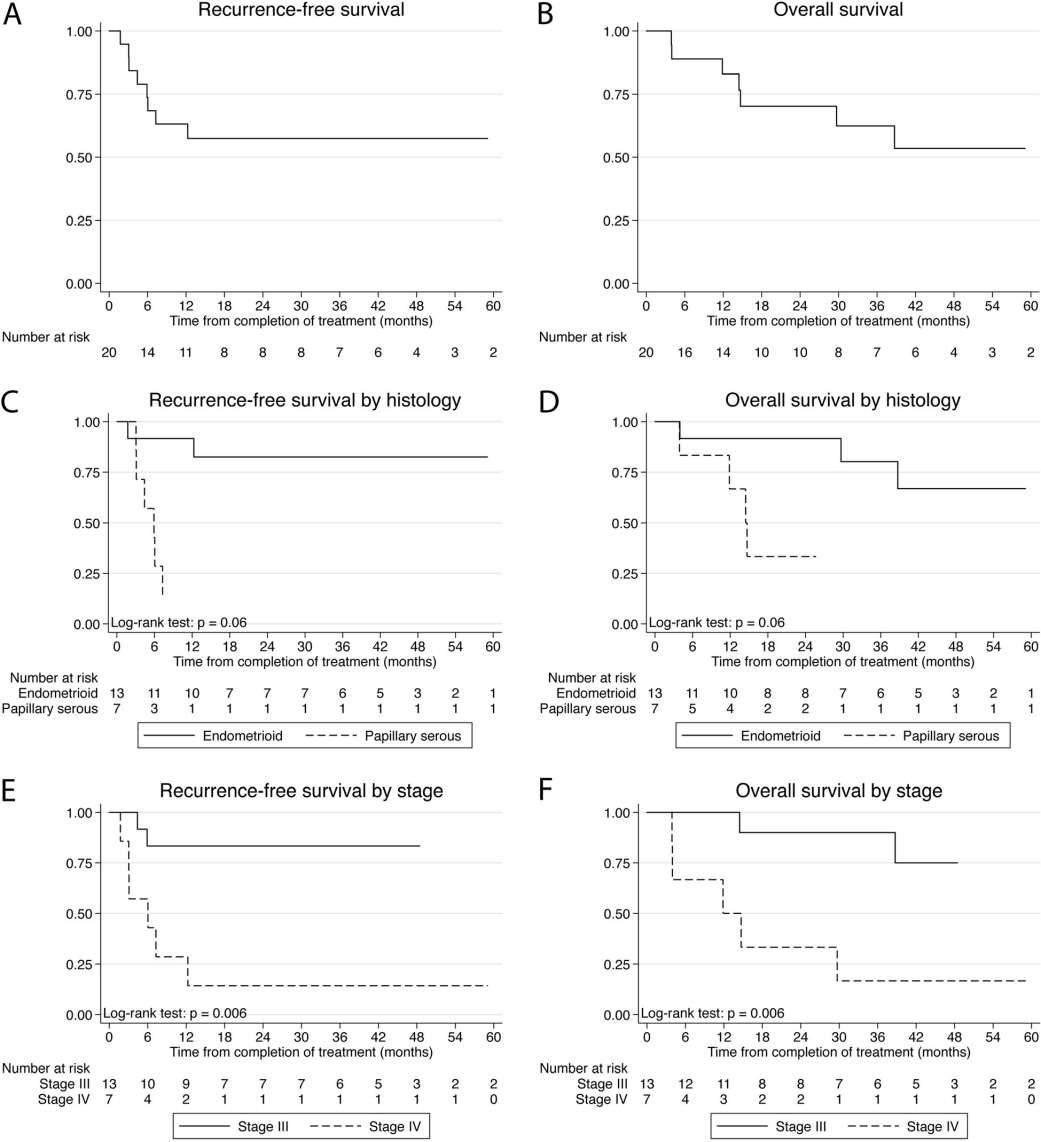
**Kaplan Meier survival estimates for 20 patients treated with consolidative whole abdomen irradiation after surgery and chemotherapy for advanced endometrial cancer. A**, **B**: recurrence-free survival (RFS) and overall survival (OS). **A**, **B**: Kaplan Meier recurrence-free survival (RFS) and overall survival (OS) estimates for 20 patients treated with consolidative whole abdomen irradiation after surgery and chemotherapy for advanced endometrial cancer. **C**, **D**: RFS and OS as a function of histology (endometriod vs. papillary serous). **E**, **F**: RFS and OS as a function of FIGO 2009 stage (stage III vs. abdomen-confined stage IV).

For the entire group of patients, KM estimates and 95% confidence intervals for RFS at 1 year were 63% (38–80%), at 3 years 57% (33-76%). KM estimates and 95% confidence intervals for OS at 1 year were 83% (56-94%), at 3 years 62% (34-81%) and at 5 years 53% (25-75%), as shown in Figure 1A-B.

Crude recurrence rates were 2/13 (15%) for EAC, 6/7 (85%) for SP, 1/7 (14%) for stage IIIA, 1/5 (20%) for stage IIIC and 6/7 (86%) for abdomen-confined stage IVB disease. None of the 9 (0%) patients with stage III EAC recurred, whereas 4/4 (100%) patients with stage IVB SP recurred. Crude recurrence rates were 2/3 (66%) for stage IVB EAC and 2/3 (66%) for stage III SP, respectively. One patient with multifocal EAC arising from endometriosis (no FIGO stage classification available) has not recurred.

All 3 suboptimally debulked patients experienced recurrence, all of them being associated with SP histology. Neither patient with residual disease found on postoperative PET-CT scan experienced a recurrence. Both had EAC.

The peritoneal cavity was the most frequent site of initial failure in the form of peritoneal carcinomatosis and malignant ascites (5/8 recurrences, 62.5%). One patient experienced a solitary vaginal cuff recurrence 4 months after end of treatments and was successfully salvaged by a laparoscopic vaginectomy. Patients with progression received various regimens of 2nd line therapy, including iv chemo, ip chemo, targeting agents within phase II studies, surgery, palliative radiotherapy of distant metastases and/or best supportive care. All recurrences occurred within a year after completion of all treatments (median: 5 months). Patterns of failure for first recurrences are shown in Table 4.

**Table 4:** Patterns of first recurrence (n = 8)

	**Distribution and%**
Localization	
Inside the abdomen only	6 (75%)
Outside the abdomen only	0 (0%)
Both in- and out the abdomen	2 (25%)
Category	
Vaginal cuff recurrence	1 (12.5%)
Peritoneal carcinomatosis	5 (62.5%)
Lymph node	4 (50%)
Pelvic, para-aortic or mesenteric	2 (25%)
Mediastinal or supraclavicular	2 (25%)
Hematogenous (liver, lung)	1 (12.5%)

On univariate Cox analysis, stage IVB and SP histology were the only statistically significant risk factors (at the p = 0.05 level) associated with worse RFS and OS, as shown in Figure 1C-F. A multivariate analysis was performed, but because of the small sample size, no statistically meaningful results were found. However, stage and histology seemed to be independently associated with OS and RFS.

## Discussion

Our data show that WAI as part of sequential multimodal based chemotherapy and radiotherapy is hematologically feasible and that acute toxicity is manageable in the context of contemporary supportive care. Tolerability was reflected in the fact that all patients completed radiotherapy treatment as planned, without toxicity-related interruptions and that no CTCAE grade 4 toxicities occurred during chemotherapy or WAI.

Systemic chemotherapy followed by WAI +/− whole pelvis boost, extended-field para-aortic boost and/or vaginal cuff High Dose Rate brachytherapy is an aggressive and potentially morbid treatment regimen. Historically, several studies have evaluated the toxicity of WAI as a consolidation treatment after chemotherapy in ovarian cancer. Myelosuppression was the cause of treatment delay in 9–36% and treatment could not be completed in 4–15% [15-19].

However, little data exist regarding combinations of chemotherapy and WAI in EC. Two approaches were reported as being feasible without excessive acute toxicity: sequential doxorubicin-cisplatin chemotherapy followed by WAI and combined radiochemotherapy with weekly cisplatin and WAI with pelvic boost and optional para-aortic irradiation [20,21]. However, a more intensive approach with radio-chemotherapy followed by systemic chemotherapy was considered not feasible due to prohibitive hematologic toxicity [22].

This is, to the best of our knowledge, the first report on feasibility of WAI after modern TP chemotherapy in endometrial cancer. A Canadian study reported on a similar treatment in ovarian cancer [15]. Overall, 34% of the 29 patients had either grade 3 or 4 acute gastrointestinal or hematologic toxicity. WAI was abandoned because of myelosuppression in 7% of patients, and toxicity-related breaks were required in 21% of patients. The results of this Canadian trial contrast with our results regarding toxicity and feasibility. This might be explained by differences in institutional practice in coping with acute toxicity under treatment. Our policy at MGH is not to interrupt radiotherapy treatment based on symptomatic side effects manageable with anti-emetics, anti-diarrheal agents and supportive I.V. hydration. Treatment is not interrupted unless absolute neutrophiles count falls below 900 cells per μL (microlitre) or platelets fall below 35,000 per μL of blood.

Late chronic radiation toxicity is a major concern for WAI, the most common reported being small bowel damage, occurring in approximately 10% of cases [19]. In the current study, there were 3 cases of small bowel obstruction, 2 of which required surgery (10%), and one case of sigmoid perforation, all in the context of tumor recurrence within the abdomen. Late hepatic toxicity (hepatic veno occlusive disease) is another rare but potential life-threatening complication following WAI [19,20]. In the present study, no patient has developed veno-occlusive disease or liver necrosis. An elevation of liver enzymes following completion of treatment was observed in 50% of our cases, mostly transient and asymptomatic, with the exception of one grade 3 transient case. Still, the inherent risk of potential lethal liver toxicity emphasizes the necessity of careful liver dose limitation. At MGH, we routinely use blocking to keep the liver dose ≤22 Gy at 1.0 Gy/fraction and kidney dose ≤18 Gy. However, with ap/pa radiotherapy technique, using blocks comes at the cost of target coverage and results in underdosed areas within the upper abdomen. Intensity-modulated radiotherapy (IMRT) has been shown to render better coverage of the entire peritoneal cavity, including the liver capsule and the diaphragm while effectively sparing the liver parenchyma, kidneys, and some bone marrow [23,24]. Future efficacy studies are likely to include this technical refinement.

We identified 20 patients treated over 11 years at MGH; this is fewer than 2 patients per year. Advanced EC is rare with few patients suitable for consolidation WAI and facilities offering WAI are even rarer. Our patients were selected because of a perceived high risk for intraperitoneal recurrence and the absence of known macroscopic residual tumor after chemotherapy. But despite clear methodological limitations of our study (small sample size, retrospective study, and modest duration of follow-up) our data shows a major and statistically significant difference in outcomes between stage III and IV and between EAC and SP. The importance of stage and histology is well documented in the literature [5-7,9,12,13]. Advanced stage EC is a heterogeneous group and the variety of therapeutic interventions in the literature - WAI alone, chemotherapy alone, multimodal treatment with or without WAI as part of the radiotherapy - makes comparisons difficult. Previous reports of treatment are summarized in Table 5.

**Table 5:** Review of literature since 2000: WAI and/or chemotherapy in patients with advanced endometrial cancer

**Authors (year)**	**No. patients**	**Treatment**	**Design**	**Outcomes**	**Comments**	**Median follow-up**
Smith RS [6]	48	WAI only	retrospective	60% 3-y FS (79% for EAC and 47% for PS/CC)	EAC: stage III-IV	37 mo.
				77% 3-y OS (89% for EAC and 68% PS/CC)	PS/CC: stage I-IV	
Stewart KD [7]	119	WAI only	prospective	Stage III 5-y OS: 67% for EAC and 40% for SP/CC	Stage III n = 81 (68%)	5.8 y
				Stage III 5-y DFS: 62% for EAC and 34% for SP/CC		
Dusenbery KE [9]	86	WAI only	retrospective	55% 5-y PFS, 46% 10-y PFS, 38% 20-y PFS	Concurrent cisplatin in 13/86	10 y
				57% 5-y OS, 48% 10-y OS, 41% 20-y OS		
Sutton G [8]	34	WAI only	prospective	38% 5-y PFS for PS	Stage I/II PS and CC only	unknown
				54% 5-y PFS for CC		
Randall ME [5]	422	Chemo vs. WAI	prospective	5-y PFS: 50% for chemo and 38% for WAI	Adjusted for stage	74 mo.
				5-y OS: 55% for chemo and 42% for WAI		
Alvarez Secord A [11]	356	Chemo vs. RT vs. multi-modality	retrospective	3-y PFS: 19% (chemo), 59% (RT), 62% (MM)	39% of WAI in RT group	38 mo.
				3-y OS: 33% (chemo), 70% (RT), 79% (MM)		
Fowler JM [20]	31	Multi-modality: chemo + WAI	prospective	53% 5-y PFS	Chemo 3 × AC	21 mo.
				60% 5-y OS		
Rochet N (present study)	20	Multi-modality: chemo + WAI	retrospective	63% 1-y, 57% 2- and 3-y RFS	TP based chemo	31.2 mo.
				83% 1-y, 70% 2-y, 62% 3-y, 53% 4-and 5-y OS		
Secord A [14]	109	Multi-modality: “sandwich” CRC vs. RC vs. CR	retrospective	3-y PFS: 69% (CRC), 47% (RC), 52% (CR)	various chemo (79% TP)	2.8 y
				3-y OS: 88% (CRC), 54% (RC), 57% (CR)		
					WAI in 13%	
Bruzzone M [12]	45	Multi-modality: chemo + pelvic RT	retrospective	30% 9-yPFS	4 × PAC	63 mo.
				53% 9-y OS	No WAI	
Geller MA [13]	42	Multi-modality: “sandwich” chemo + pelvic +/− EFRT	prospective	87% 1-y PFS, 71% 3-y PFS, 64% 5-y PFS	6 × TP	28 mo.
					No WAI	
				95% 1-y OS, 90% 3-y OS, 71% 5-y OS		

*Abbreviations*: *No*. Number of, *WAI* Whole abdomen radiotherapy, *Chemo* Chemotherapy, *EFRT* Extended-fields radiotherapy, *EAC* Endometrioid adenocarcinoma, *PS* Papillary serous, *CC* Clear cell, *DFS* Disease-free survival, *PFS* Progression-free survival, *OS* Overall survival, *RFS* Recurrence-free survival, *MM* Multi-modality, *CRC* Chemotherapy, interval radiotherapy, subsequent chemotherapy, *RC* Radiotherapy followed by chemotherapy, *CR* Chemotherapy followed by radiotherapy, *RT* Radiotherapy, *TP* Carboplatin/paclitaxel, *PAC* Cisplatin/doxorubicin/cytoxan, *AC* Doxorubicin/cisplatin.

In our study, despite the maximum effort given to the abdomen, the peritoneal cavity was still the most frequent site of initial failure, with 5/8 recurrences, 2 of which were associated with additional sites. There are areas of potential underdosage in the upper abdomen when using liver and kidney shielding. IMRT has the potential for more homogeneous target coverage and for conformal dose escalation and might decrease the intra-abdominal recurrence rate [23,24]. However, this theoretical benefit has not yet been clinically proven.

The fact that 2 of 20 patients considered optimally debulked were found with FDG-avid residual disease on postoperative PET/CT using contrast enhanced diagnostic quality CT suggests a need for preoperative imaging in selected patients [25].

The curative potential of WAI as only postoperative adjuvant therapy has been proven for stage III and abdomen-confined IVB, especially for EAC [10]. However, the role of WAI in the era of polyagent chemotherapy treatment remains controversial [12-14].

## Conclusions

In summary, we demonstrate that in the contemporary era, WAI after carboplatin-paclitaxel based chemotherapy is feasible and safe with both manageable acute and late toxicities. This approach was associated with favorable outcomes in our patients with EAC, possibly attributable to radiotherapy benefit. Based on feasibility and manageable toxicity, it is our hypothesis that WAI coordinated with chemotherapy may warrant prospective investigation in this patient population. Given the challenges of accrual, an international study would be needed. Patients with SP histology did poorly and did not benefit from consolidative treatment as delivered in this study.

## Competing interest

Actual or potential conflicts of interest do not exist. All authors declare that there are no conflicts of interest. NR is financially supported by a scholarship from the German Society of Radiation Oncology (DEGRO) for this project. This study has been presented as a poster during ASTRO’s 2012 Annual Meeting in Boston (Abstract #2617).

## Authors’ contributions

All authors have read and approved the manuscript and agree to its submission. This manuscript has not been previously published.

## References

[B1] Siegel R Naishadham D Jemal A Cancer statistics, 2012 CA Cancer J Clin 2012 62 10 29 10.3322/caac.20138 22237781

[B2] Ueda SM Kapp DS Cheung MK Trends in demographic and clinical characteristics in women diagnosed with corpus cancer and their potential impact on the increasing number of deaths Am J Obstet Gynecol 2008 198 218 6 18226630 10.1016/j.ajog.2007.08.075

[B3] Goff BA Goodman A Muntz HG Surgical stage IV endometrial carcinoma: a study of 47 cases Gynecol Oncol 1994 52 237 240 10.1006/gyno.1994.1038 8314145

[B4] Onda T Yoshikawa H Mizutani K Treatment of node-positive endometrial cancer with complete node dissection, chemotherapy and radiation therapy Br J Cancer 1997 75 836 1841 10.1038/bjc.1997.313 PMC2223619 9192991

[B5] Randall ME Filiaci VL Muss H Randomized phase III trial of whole-abdominal irradiation versus doxorubicin and cisplatin chemotherapy in advanced endometrial carcinoma: a gynecologic oncology group study J Clin Oncol 2006 4 36 44 16330675 10.1200/JCO.2004.00.7617

[B6] Smith RS Kapp DS Chen Q Treatment of high-risk uterine cancer with whole abdominopelvic radiation therapy Int J Radiat Oncol Biol Phys 2000 48 767 778 10.1016/S0360-3016(00)00724-0 11020574

[B7] Stewart KD Martinez AA Weiner S Ten-year outcome including patterns of failure and toxicity for adjuvant whole abdominopelvic irradiation in high-risk and poor histologic feature patients with endometrial carcinoma Int J Radiat Oncol Biol Phys 2002 54 527 535 10.1016/S0360-3016(02)02947-4 12243832

[B8] Sutton G Axelrod JH Bundy BN Adjuvant whole abdominal irradiation in clinical stages I and II papillary serous or clear cell carcinoma of the endometrium: a phase II study of the gynecologic oncology group Gynecol Oncol 2006 100 349 354 10.1016/j.ygyno.2005.08.037 16213007

[B9] Dusenbery KE Potish RA Gold DG Utility and limitations of abdominal radiotherapy in the management of endometrial carcinomas Gynecol Oncol 2005 96 635 642 10.1016/j.ygyno.2004.11.048 15721405

[B10] Lee SW Russell AH Kinney WK Whole abdomen radiotherapy for patients with peritoneal dissemination of endometrial adenocarcinoma Int J Radiat Oncol Biol Phys 2003 56 788 792 10.1016/S0360-3016(03)00066-X 12788186

[B11] Alvarez SA Havrilesky LJ Bae-Jump V The role of multi-modality adjuvant chemotherapy and radiation in women with advanced stage endometrial cancer Gynecol Oncol 2007 107 285 291 10.1016/j.ygyno.2007.06.014 17688923

[B12] Bruzzone M Miglietta L Franzone P Combined treatment with chemotherapy and radiotherapy in high-risk FIGO stage III-IV endometrial cancer patients Gynecol Oncol 2004 93 345 352 10.1016/j.ygyno.2004.02.008 15099944

[B13] Geller MA Ivy JJ Ghebre R A phase II trial of carboplatin and docetaxel followed by radiotherapy given in a “sandwich” method for stage III, IV, and recurrent endometrial cancer Gynecol Oncol 2011 121 112 117 10.1016/j.ygyno.2010.12.338 21239048 PMC6231578

[B14] Secord AA Havrilesky LJ O’Malley DM A multicenter evaluation of sequential multimodality therapy and clinical outcome for the treatment of advanced endometrial cancer Gynecol Oncol 2009 114 442 447 10.1016/j.ygyno.2009.06.005 19560193

[B15] Dinniwell R Lock M Pintilie M Consolidative abdominopelvic radiotherapy after surgery and carboplatin/paclitaxel chemotherapy for epithelial ovarian cancer Int J Radiat Oncol Biol Phys 2005 62 104 110 10.1016/j.ijrobp.2004.09.010 15850909

[B16] Fyles AW Dembo AJ Bush RS Analysis of complications in patients treated with abdomino-pelvic radiation therapy for ovarian carcinoma Int J Radiat Oncol Biol Phys 1992 22 847 851 10.1016/0360-3016(92)90778-G 1555975

[B17] Pickel H Lahousen M Petru E Consolidation radiotherapy after carboplatin-based chemotherapy in radically operated advanced ovarian cancer Gynecol Oncol 1999 72 215 219 10.1006/gyno.1998.5184 10021304

[B18] Sorbe B Consolidation treatment of advanced (FIGO stage III) ovarian carcinoma in complete surgical remission after induction chemotherapy: a randomized, controlled, clinical trial comparing whole abdominal radiotherapy, chemotherapy, and no further treatment Int J Gynecol Cancer 2003 13 278 286 10.1046/j.1525-1438.2003.13193.x 12801256

[B19] Whelan TJ Dembo AJ Bush RS Complications of whole abdominal and pelvic radiotherapy following chemotherapy for advanced ovarian cancer Int J Radiat Oncol Biol Phys 1992 22 853 858 10.1016/0360-3016(92)90779-H 1555976

[B20] Fowler JM Brady WE Grigsby PW Sequential chemotherapy and irradiation in advanced stage endometrial cancer: a gynecologic oncology group phase I trial of doxorubicin-cisplatin followed by whole abdomen irradiation Gynecol Oncol 2009 112 553 557 10.1016/j.ygyno.2008.11.026 19135232

[B21] Reisinger SA Asbury R Liao SY A phase I study of weekly cisplatin and whole abdominal radiation for the treatment of stage III and IV endometrial carcinoma: a gynecologic oncology group pilot study Gynecol Oncol 1996 63 299 303 10.1006/gyno.1996.0326 8946862

[B22] Soper JT Reisinger SA Ashbury R Feasibility study of concurrent weekly cisplatin and whole abdominopelvic irradiation followed by doxorubicin/cisplatin chemotherapy for advanced stage endometrial carcinoma: a gynecologic oncology group trial Gynecol Oncol 2004 95 95 100 10.1016/j.ygyno.2004.06.041 15385116

[B23] Rochet N Sterzing F Jensen A Helical tomotherapy as a new treatment technique for whole abdominal irradiation Strahlenther Onkol 2008 184 145 149 10.1007/s00066-008-1772-z 18330510

[B24] Rochet N Sterzing F Jensen AD Intensity-modulated whole abdominal radiotherapy after surgery and carboplatin/taxane chemotherapy for advanced ovarian cancer: phase I study Int J Radiat Oncol Biol Phys 2010 76 1382 1389 10.1016/j.ijrobp.2009.03.061 19628341

[B25] Haie-Meder C Mazeron R Magne N Clinical evidence on PET-CT for radiation therapy planning in cervix and endometrial cancers Radiother Oncol 2010 96 351 355 10.1016/j.radonc.2010.07.010 20709417

